# In Situ ZrB_2_ Formation in B_4_C Ceramics and Its Strengthening Mechanism on Mechanical Properties

**DOI:** 10.3390/ma15227961

**Published:** 2022-11-10

**Authors:** Wankai Yao, Junbing Yan, Xiangcheng Li, Pingan Chen, Yingli Zhu, Boquan Zhu

**Affiliations:** 1 The State Key Laboratory of Refractories and Metallurgy, Wuhan University of Science and Technology, Wuhan 430081, China; 2 Wuhan Xinxin Semiconductor Manufacturing Co., Ltd., Wuhan 430223, China

**Keywords:** spark plasma sintering (SPS), boron carbide (B_4_C), Zirconium Diboride (ZrB_2_), reaction sintering

## Abstract

In order to reduce the sintering temperature and improve the mechanical properties of B_4_C ceramics, ZrB_2_ was formed in situ using the SPS sintering method with ZrO_2_ and B_4_C as raw materials. Thermodynamic calculations revealed that CO pressure affected the formation of ZrB_2_ at temperatures from 814 °C to 1100 °C. The experimental results showed that the ZrB_2_ grain size was <5 µm and that the grains were uniformly distributed within the B_4_C ceramics. With an increase in ZrO_2_ content, the Vickers hardness and flexural strength of the B_4_C ceramics first increased and then decreased, while the fracture toughness continuously increased. When the content of ZrO_2_ was 15 wt%, the Vickers hardness, fracture toughness and flexural strength of B_4_C ceramics were 35.5 ± 0.63 GPa, 3.6 ± 0.24 MPa·m^1/2^ and 403 ± 10 MPa, respectively. These results suggest that ZrB_2_ inhibits B_4_C grain growth, eliminates crack tip stress, and provides fine grain to strengthen and toughen B_4_C ceramics.

## 1. Introduction

Due to their low density, high hardness and high elastic modulus, boron carbide (B_4_C) ceramics have been used in the manufacturing of armor, cutting tools and neutron absorbers [1,2,3,4,5]. However, further application of B_4_C ceramics is limited by the high sintering temperature (>2000 °C) and low fracture toughness (*K*_IC_ < 2.2 MPa·m^1/2^), which can be attributed to the strong B-C covalent bonds, low atomic diffusion coefficient and low dislocation plasticity [6,7,8,9,10]. Therefore, it is essential to lower the sintering temperature and improve the mechanical properties of B_4_C ceramics.

The addition of second phases, such as SiC, Al_2_O_3_, TiC and TiO_2_, is an effective method of reducing the sintering temperature and improving the mechanical properties of B_4_C ceramics [11,12], but ultimately results in reduced material hardness. Moreover, brittle compounds are formed during the densification process, leading to a low fracture toughness. Transitional metal diborides (TiB_2_, ZrB_2_) are useful additives which have similar properties to B_4_C ceramics [13,14,15]. Among them, Zirconium Diboride (ZrB_2_) has a high melting point (3245 °C), high hardness (14–23 GPa) and high flexural strength (higher than 500 MPa), which could improve the flexural strength and fracture toughness of B_4_C ceramics [16,17].

Many reports have shown that the addition of ZrB_2_ could efficiently enhance the mechanical properties of B_4_C ceramics. For example, He et al. [18] found that ZrB_2_ promoted the sintering process and increased the ceramic’s high critical crack size, resulting in excellent thermal shock resistance. In order to inhibit the grain growth of B_4_C, Lin et al. [19] prepared B_4_C ceramics by pressureless sintering using ZrO_2_ as a sintering aid. The in situ formation of ZrB_2_ inhibited the abnormal growth of B_4_C grains, prolonged the crack extension path and improved the sintering activity. Guo et al. [20] prepared B_4_C-ZrB_2_ ceramics at low temperature, resulting in an average B_4_C grain size of 1–2 μm. The hardness and fracture toughness reached 33.0 ± 1.3 GPa and 4.1 ± 0.2 MPa·m^1/2^, respectively. Rehman et al. [21] prepared B_4_C ceramics by SPS at 1700 °C; the average grain size of ZrB_2_ was 1.3 ± 0.2 μm. The Vickers hardness, fracture toughness and flexural strength were 31.28 GPa, 4.2 MPa·m^1/2^ and 511 MPa, respectively. However, the role of ZrB_2_ as a sintering aid and grain growth inhibitor requires further investigation, and the effect of ZrB_2_ on the fracture mode transformation of B_4_C ceramics also needs to be clarified.

In this work, we aim to obtain densified B_4_C ceramics with the in situ formation of ZrB_2_ at 1700 °C using the spark plasma sintering (SPS) method, with ZrB_2_ grains homogeneously distributed in the B_4_C ceramics. The reaction between ZrO_2_ and B_4_C could effectively reduce the sintering temperature, and the pinning effect of ZrB_2_ is expected to inhibit the B_4_C grain growth, achieving desirable mechanical properties.

## 2. Materials and Methods

### 2.1. Materials and Processes

B_4_C powder (1 μm, analytically pure) and nano-ZrO_2_ (50 nm, purity > 99.9%) were used as raw materials. Nano-Y_2_O_3_ (50 nm, purity > 99.9%) was used as a sintering aid.

The compositions of the samples are shown in Table 1. The raw materials were mixed with stainless steel balls in anhydrous ethanol with a ball-to-material ratio of 5:1 and a mixing time of 6 h at 250 r/min. After mixing, the mixtures were dried in a drying oven at 80 °C for 24 h and then passed through a 200-mesh sieve. Then, the mixtures were sintered by SPS (Dr. Sinter-3.20 MKII, Sumitomo, Tokyo, JPN) under vacuum. The heating rate was maintained at 100 °C/min and a uniaxial load of 5 kN was applied during the heating stage to ensure good contact conductivity with the graphite indenter. After the temperature rose to 1600 °C, the uniaxial load was slowly increased to 15 kN. Then, the sintering temperature was increased to 1700 °C for 6 min. The sintered ceramics were cooled in the furnace to about 150 °C and the samples were taken out to obtain B_4_C ceramics.

### 2.2. Characterizations

The bulk density of B_4_C ceramics was measured using the Archimedes method. The Vickers hardness and fracture toughness were measured by the indentation method using a Vickers hardness tester (HV-50A, Huayin, Laizhou, China). The spacing between each indentation was ensured to be greater than 100 μm to avoid crack deformation or the overlapping of extended areas caused by indentation. Five effective indentations were also used to calculate the mean and error of Vickers hardness and fracture toughness. The B_4_C ceramics were processed into long strip samples of 2 mm × 3 mm × 20 mm using a diamond wire cutting machine, and the bending strength of the samples was measured using a three-point bending test with a span of 16 mm and a loading rate of 0.5 mm/min. The mean values of flexural strength and error analyses were calculated with 3 sets of valid data. The phase compositions were characterized by X-ray diffractometer (XRD, X’Pert MPD Pro, Philips, Amsterdam, The Netherlands). The voltage and current of the Cu Kα1 radiation were 40 kV and 40 mA, respectively. The scanning range (2*θ*) was 10°–90°. The microscopic morphologies were characterized using a field-emission scanning electron microscope (FESEM, Nova NanoSem 400, Thermo, Waltham, MA, USA).

## 3. Results and Discussion

### 3.1. Thermodynamic Calculations

The in situ generation of ZrB_2_ from B_4_C and nano-ZrO_2_ is represented in Reaction (1):

(1) $$B_{4}C(s)+2{\mathrm{ZrO}}_{2}(s)+3C(s)\to 2{\mathrm{ZrB}}_{2}(s)+4\mathrm{CO}(g)$$

**Figure 1:**
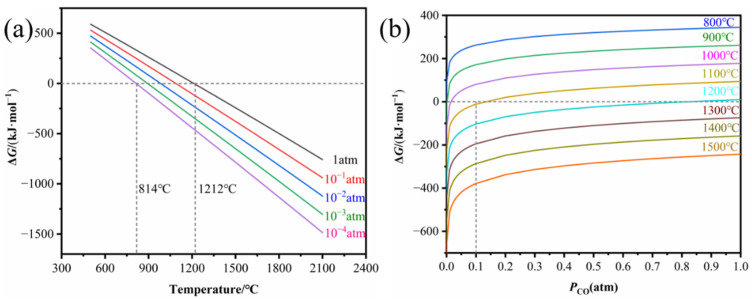
Gibbs’ free energy change of reaction (1). (**a**) Variation in temperature at different *P*_CO_; (**b**) variation with *P*_CO_ at different temperatures.

According to thermodynamic calculations by FactSage, the variation in Gibbs’ free energy of reaction (1) as a function of temperature at different *P*_CO_ (pressure of CO) and as a function of *P*_CO_ at different temperatures is shown in Figure 1. Figure 1a shows that in the standard state (*P*_CO_ = 0.986 atm), a temperature above 1212 °C is favorable for the formation of ZrB_2_. It is expected that reaction (1) proceeds more rapidly when the reaction is carried out under vacuum. Therefore, pure ZrB_2_ can be synthesized at a temperature as low as 814 °C when *P*_CO_ = 10^−5^ atm, according to Figure 1a. However, the CO generated by the sample surrounded by graphite die cannot be expelled in time, leading to *P*_CO_ > 10^−4^ atm; thus, the reaction will proceed at a temperature higher than 814 °C. Figure 1b further illustrates that reaction (1) needs to proceed at temperatures above 1100 °C when *P*_CO_ is in the range of 0–0.1 atm. In the experiments, the vacuum pump worked continuously and the vacuum level in the furnace was as low as 8 × 10^−5^ atm during SPS, maintaining the *P*_CO_ at a low level and thus promoting the low-temperature densification of B_4_C ceramics.

### 3.2. XRD Phase Analysis

**Figure 2:**
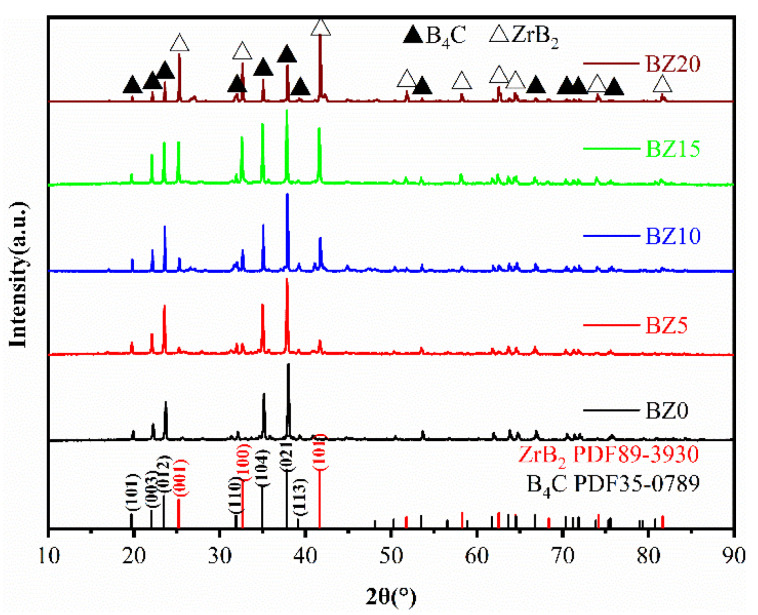
XRD patterns of B_4_C ceramics with different nano-ZrO_2_ contents.

The XRD patterns of B_4_C ceramics prepared by SPS with different ZrB_2_ contents are shown in Figure 2. The ceramics are composed of B_4_C and ZrB_2_, and no residual nano-ZrO_2_ phase can be detected, indicating that nano-ZrO_2_ and B_4_C completely reacted to form the ZrB_2_ phase. The B_4_C in the samples all had the rhombohedral crystal structure (PDF #35-0798). The diffraction peaks of samples at 2*θ* of 21.21°, 32.64°, and 41.69° were all taken from the ZrB_2_ crystal (PDF #89-3930), and correspond to the (110), (200) and (211) crystal planes of its hexagonal crystal structure, respectively. The intensity of the ZrB_2_ diffraction peak increases with the addition of nano-ZrO_2_, while the intensity of the B_4_C diffraction peak is the opposite, which is consistent with the expected results. The XRD results indicate that nano-ZrO_2_ can react with B_4_C in situ to form ZrB_2_, which can promote the diffusion effect between B_4_C particles during sintering and achieve the densification of B_4_C ceramics.

### 3.3. Microstructural Analysis

**Figure 3:**
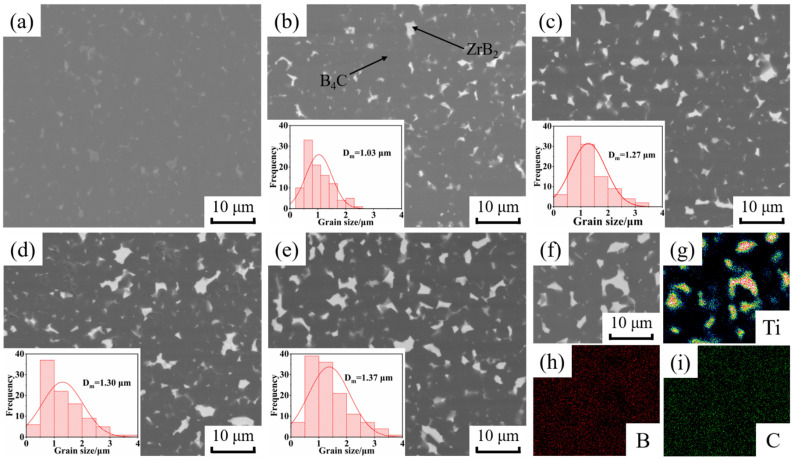
BSE images of B_4_C ceramics and grain size of ZrB_2_. (**a**) BZ0, (**b**) BZ5, (**c**) BZ10, (**d**) BZ15, (**e**) BZ20 (**f**) BSE image of BZ15; element of Ti (**g**), B (**h**) and C (**i**).

The BSE images of B_4_C ceramics and the grain size of ZrB_2_ prepared by SPS are shown in Figure 3. The distribution of B, C and Ti elements in sample BZ15 are shown in Figure 3f–i. Combined with XRD, it can be deduced that the dark gray phase is B_4_C, and the bright white phase is ZrB_2_. Figure 3a shows that the addition of 3 wt% nano-Y_2_O_3_ can achieve a polished surface of B_4_C ceramics with no obvious pores without the addition of nano-ZrO_2_. Figure 3b–e show that the amount of ZrB_2_ produced gradually increases as the addition of nano-ZrO_2_ increases from 5 wt% to 20 wt%, and the average particle size of ZrB_2_ increases from 1.03 μm to 1.37 μm. The in situ reaction of nano-ZrO_2_ to generate ZrB_2_ facilitates the flowability between B_4_C particles and promotes the densification and pore discharge of B_4_C ceramics, such that there are no obvious pores on the polished surface. In addition, ZrB_2_ not only has small grain size, but is also uniformly distributed within the B_4_C ceramics, which will effectively improve the mechanical properties.

**Figure 4:**
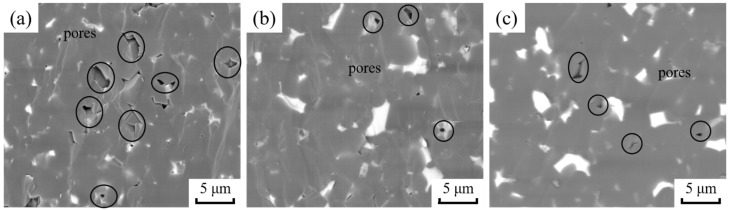
BSE diagrams of B_4_C ceramics’ fracture morphology. (**a**) BZ0; (**b**) BZ10; (**c**) BZ20.

BSE diagrams of the fracture morphology of B_4_C ceramics with different nano-ZrO_2_ contents are shown in Figure 4. The fracture mode is mainly through crystal fracture, and the fracture surface is relatively neat and smooth. This indicates a favorable degree of connection between the grains after SPS. In addition, there are more pores in the BZ0 sample, but the number of pores decreases significantly with the addition of nano-ZrO_2_. This indicates that nano-ZrO_2_ promotes the densification of B_4_C ceramics.

**Figure 5:**
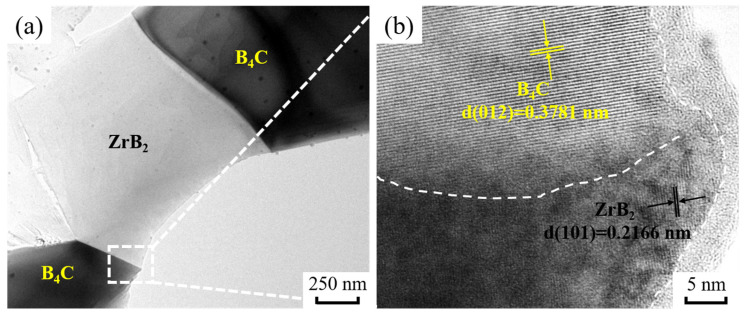
TEM diagrams of BZ15. (**a**) TEM; (**b**) HRTEM.

TEM images of BZ15 are shown in Figure 5. Figure 5a shows the interface between B_4_C and ZrB_2_ in the B_4_C ceramics. The HRTEM image shown in Figure 5b indicates that the grain boundary between B_4_C (d = 0.3781 nm, (012) zone axis) and ZrB_2_ (d = 0.2166 nm, (101) zone axis) is clean. This result indicates that the grain boundary strength between B_4_C and ZrB_2_ is strong. It is well-known that a high binding force between the matrix phase and second phase has a significant effect on mechanical properties. Therefore, this suggests that excellent mechanical properties can be obtained from these B_4_C ceramic samples.

### 3.4. Mechanical Properties of the B_4_C Ceramics

**Figure 6:**
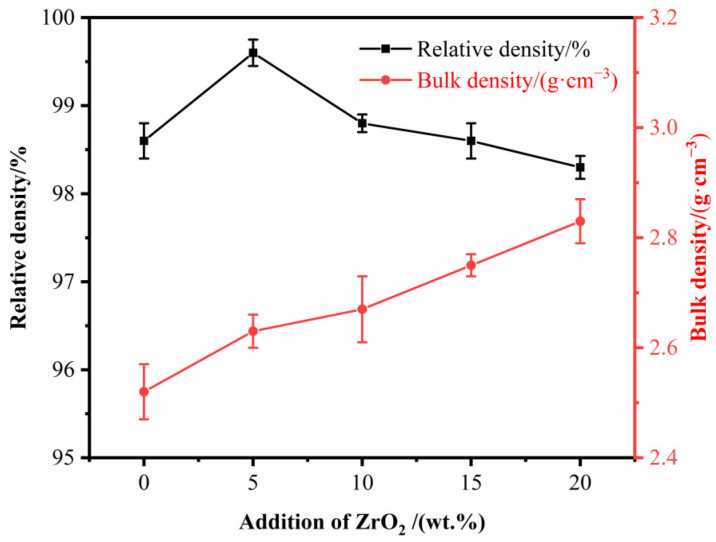
Relative density and bulk density of B_4_C ceramics at different ZrB_2_ contents.

The relative density and bulk density of B_4_C ceramics prepared by SPS with different ZrO_2_ contents are shown in Figure 6. The relative densities measured are all above 98.3%, and increase and then decrease with increasing ZrO_2_ content. The bulk density continues to increase, between 2.52 g/cm^3^ and 2.83 g/cm^3^. The relative density reaches a maximum of 99.6% when the content of ZrO_2_ is 5 wt%; this can be attributed to the in situ reaction of nano-ZrO_2_ to generate ZrB_2_, which that promotes the flowability between B_4_C particles and obtains dense ceramics. When the content of ZrO_2_ is higher than 5 wt%, the CO gas generated by the sintering reaction also increases, causing the relative density to decrease. The observed increase in bulk density can be explained by the fact that the theoretical density of ZrB_2_ (6.1 g/cm^3^) is greater than that of B_4_C, meaning that an increase in ZrB_2_ content leads to a gradual increase in the bulk density of the ceramics.

**Figure 7:**
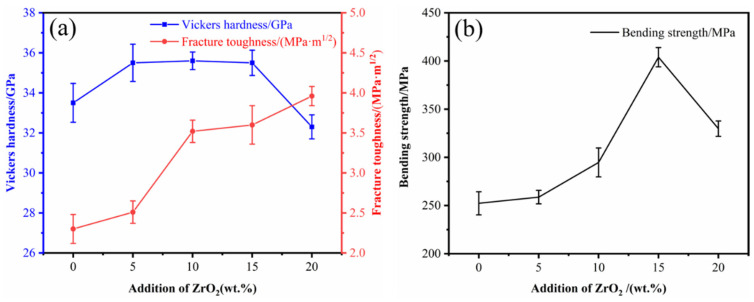
Mechanical properties of B_4_C ceramics with different nano-ZrO_2_ contents. (**a**) Vickers hardness and fracture toughness; (**b**) bending strength.

The Vickers hardness, fracture toughness and flexural strength of B_4_C ceramics prepared by SPS at different ZrO_2_ contents are shown in Figure 7. From Figure 7a, it can be seen that with the increase of ZrO_2_ content, the Vickers hardness shows a trend of first increasing and then decreasing, and the fracture toughness gradually increases. The Vickers hardness is maintained above 35.5 GPa with a of ZrO_2_ content of 5–15 wt%, but decreases to 32.3 GPa with a ZrO_2_ content of 20 wt%. Fracture toughness gradually increases from 2.3 MPa·m^1/2^ (BZ0) to 3.96 MPa·m^1/2^ (BZ20). This is because an increase in the relative density of B_4_C ceramics leads to an increase in Vickers hardness, but the presence of pores can also have a negative effect on Vickers hardness. The ZrB_2_ generated in situ from nano-ZrO_2_ has a pinning effect, which inhibits the growth of B_4_C particles. At the same time, the residual stresses present during cooling can block the crack expansion of B_4_C ceramics due to the difference in thermal expansion coefficients of B_4_C and ZrB_2_. Both processes are beneficial for the improvement of the fracture toughness of B_4_C ceramics. As can be seen in Figure 7b, the bending strength of the B_4_C ceramics shows a trend of increasing and then decreasing with an increase in ZrO_2_ content. The bending strength reaches a maximum value of 404 MPa at 15 wt% ZrO_2_, which is 60.3% higher than the BZ0 sample (252 MPa). The addition of nano-ZrO_2_ promotes the sintering between B_4_C particles and inhibits their further growth. Furthermore, the bending strength of ZrB_2_ is greater than that of B_4_C, ultimately improving the bending strength of the B_4_C ceramics. However, when the content of ZrO_2_ reaches 20 wt%, the generation of CO gas leads to an increase in the number of pores and a decrease in the density, which results in a lower bending strength. When the content of ZrO_2_ is 15 wt%, the Vickers hardness, fracture toughness and bending strength of sample BZ15 are 35.5 GPa, 3.6 MPa·m^1/2^ and 404 MPa, respectively, with optimal overall performance.

### 3.5. Densification and Toughening Mechanism Analysis

**Figure 8:**
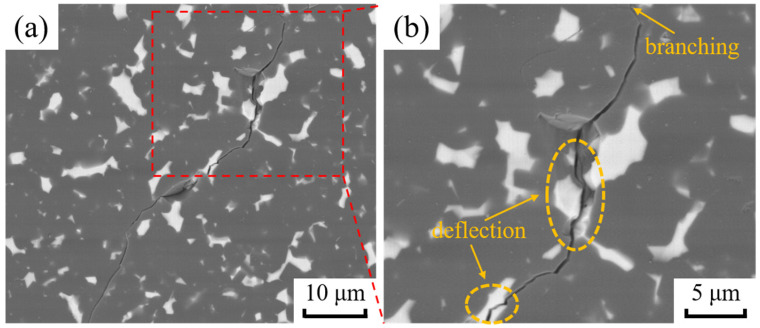
BSE diagrams of the crack expansion path of sample BZ15. (**a**) Partial view; (**b**) Partial enlarged view.

Crack propagation within the BZ15 sample following the Vickers hardness indentation tests is shown in Figure 8. There are obvious branching and deflection phenomena in crack extension, which can increase the energy required for crack extension and thus improve the fracture toughness of B_4_C ceramics. In addition, the fracture mode of the B_4_C ceramics changes from a traditional transgranular fracture to a hybrid mode of intergranular and transgranular fracture, which is beneficial for the improvement of fracture toughness.

The different coefficients of thermal expansion between the matrix and the second phase in ceramics could induce residual stress during the cooling process. The effect of this is more obvious when the strength and elastic modulus of the second phase are higher than those of the matrix. Since the thermal expansion coefficients of B_4_C and ZrB_2_ are quite different, the interface between ZrB_2_ and B_4_C will therefore be subject to residual stress (*p*_i_) during the cooling process [22].

(2) $$p_{i}=\frac{\Delta \alpha \Delta T}{\frac{1+\nu_{m}}{2E_{m}}+\frac{1-2\nu_{p}}{E_{p}}}$$

where Δ*α* is the difference between the coefficients of thermal expansion of B_4_C and ZrB_2_, Δ*T* is the temperature difference between the cooling stages of SPS, *ν* is Poisson’s ratio, *E* is elastic modulus, and the subscripts *p* and *m* stand for ZrB_2_ and B_4_C, respectively.

In this condition, *σ*_r_ (radial stress) and *σ*_t_ (tangential stress) are generated in the matrix at a distance *r* from the particles [22].

(3) $$\sigma r=-pi{\left(\frac{R}{r}\right)}^{3}$$

and

(4) $$\sigma t=\frac{1}{2}pi{\left(\frac{R}{r}\right)}^{3}$$

where *R* is the radius of the particle and *r* is the distance from a point in the stress field to the center of the particle. It can be seen that both *σ*_r_ and *σ*_t_ are proportional to (*R*/*r*)^3^.

**Figure 9:**
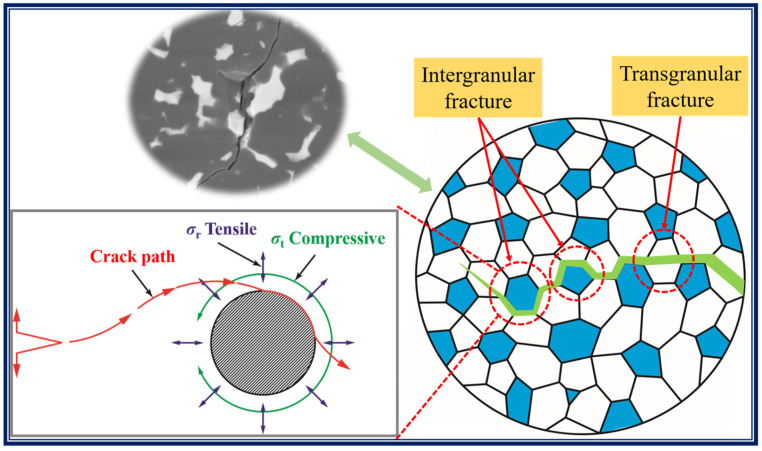
Schematic diagram of residual stress and crack extension in B_4_C ceramics.

When a crack passes through this stress field, a deflection will occur, as shown in Figure 9. When second phase particles are encountered ahead of the crack, the radial tensile stress *σ*_r_ increases in the matrix (*σ*_r_ is maximal at the interface), causing the crack to deflect in the direction of the particles and reach the particle/matrix interface before propagating along the original extension direction. The combined effect of *σ*_r_ and *σ*_t_ lengthens the crack expansion path in the matrix and consumes the energy for crack expansion. Therefore, the crack deflection caused by the residual stress field produces an absolute toughening effect. Figure 8 shows that the crack extension undergoes deflection and bypass, and that there is a secondary crack generation on the primary crack, which increases the path of crack extension and consumes the energy of crack extension. If the crack propagation path is more tortuous, and the volume fraction of the second phase component which leads to crack deflection is higher, then the effect of crack deflection and toughening will be significant.

### 3.6. Comparison of Properties of B_4_C Ceramics in Recent Years

**Figure 10:**
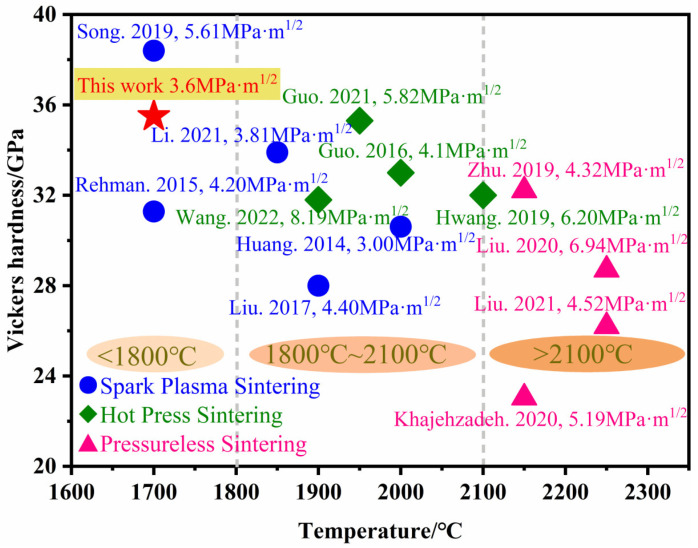
Comparison of the performance of B_4_C ceramics in recent years [20,21,23,24,25,26,27,28,29,30,31,32,33].

The Vickers hardness and fracture toughness of B_4_C ceramics prepared under different sintering methods in recent years are shown in Figure 10 [20,21,23,24,25,26,27,28,29,30,31,32,33]. The different sintering methods described are pressureless sintering, hot-pressure sintering and spark plasma sintering. Although the Vickers hardness and fracture toughness of B_4_C ceramics are high, the sintering temperature of dense B_4_C ceramics is almost above 1800 °C. In this study, B_4_C ceramics are successfully prepared at 1700 °C by in situ reaction using nano-ZrO_2_ as a sintering aid. The maximum values of Vickers hardness and fracture toughness are 35.5 GPa and 3.6 MPa·m^1/2^, respectively. The in situ-generated ZrB_2_ inhibits the growth of B_4_C grains, deflecting and bifurcating the cracks, thus enhancing the mechanical properties of B_4_C ceramics.

The phase-field model of Roy shows that interfacial stress is an important factor affecting the interfacial stress distribution at interfaces and the phase-field solution [34]. Therefore, the effect of interfacial stress between B_4_C and ZrB_2_, as well as the ZrO_2_ phase transition process on the evolution of the nano-microstructure, must be studied in further detail. In addition, Mahdi investigated the effect of high pressure on the phase transformation [35], which may offer some insight into the effect of residual stress on ZrB_2_ grain growth due to the difference in the coefficients of thermal expansion between B_4_C and ZrB_2_.

## 4. Conclusions

In this study, B_4_C ceramics were prepared by the in situ generation of ZrB_2_ using SPS at low temperature. The effects of ZrB_2_ on the microstructure and mechanical properties of the B_4_C ceramics were characterized and tested. The following conclusions were drawn.

(1) The ceramics were composed of B_4_C and ZrB_2_. The nano-ZrO_2_ reacted to form fine-crystalline ZrB_2_, which was uniformly distributed in the B_4_C ceramics. The in situ generation of ZrB_2_ contributed to the flowability between B_4_C particles, improving the sintering activity and achieving the densification of the B_4_C ceramics at low temperature.

(2) The relative density, Vickers hardness and bending strength of the B_4_C ceramics first increased and then decreased with the increase of ZrB_2_ content, and the bulk density and fracture toughness increased continuously. The relative density was above 98.3% and the bulk density was 2.52–2.83 g/cm^3^. When the content of ZrO_2_ was 15 wt%, the Vickers hardness, fracture toughness and bending strength were 35.5 ± 0.63 GPa, 3.6 ± 0.24 MPa·m^1/2^ and 403 ± 10 MPa, respectively, with the best overall performance.

(3) The pinning effect of ZrB_2_ particles changed the fracture mode of the B_4_C ceramics from traditional transgranular fracture to a hybrid mode of intergranular and transgranular fracture, which improved the bending strength of the ceramics. The high bonding strength between powders and the residual stress between B_4_C and ZrB_2_ led to crack deflection and crack branching during the crack propagation, which resulted in the high mechanical properties of the B_4_C ceramics.

## Figures and Tables

**Table 1:** Compositions of samples.

Samples	BZ0	BZ5	BZ10	BZ15	BZ20
B_4_C/wt%	100	95	90	85	80
ZrO_2_/wt%	0	5	10	15	20
Y_2_O_3_/wt%	3	3	3	3	3

## Data Availability

All data generated or analyzed during this study can be made available by the corresponding author upon request.

## References

[1] Sha W., Liu Y., Zhou Y., Huang Y., Huang Z. (2022). Effect of Carbon Content on Mechanical Properties of Boron Carbide Ceramics Composites Prepared by Reaction Sintering. Materials.

[2] Reddy K.M., Guo D., Song S., Cheng C., Han J., Wang X., An Q., Chen M. (2021). Dislocation-mediated shear amorphization in boron carbide. Sci. Adv..

[3] Thévenot F. (1990). Boron carbide—A comprehensive review. J. Eur. Ceram. Soc..

[4] Chang Y., Sun X., Ma M., Mu C., Li P., Li L., Li M., Nie A., Xiang J., Zhao Z. (2020). Application of hard ceramic materials B_4_C in energy storage: Design B_4_C@ C core-shell nanoparticles as electrodes for flexible all-solid-state micro-supercapacitors with ultrahigh cyclability. Nano Energy.

[5] Qu Z., Yu C., Wei Y., Su X., Du A. (2022). Thermal conductivity of boron carbide under fast neutron irradiation. J. Adv. Ceram..

[6] Domnich V., Reynaud S., Haber R.A., Chhowalla M. (2011). Boron carbide: Structure, properties, and stability under stress. J. Am. Ceram. Soc..

[7] Zan Y., Zhou Y., Zhao H., Liu Z., Wang Q., Wang D., Wang W., Xiao B., Ma Z. (2020). Enhancing high-temperature strength of (B_4_C+ Al_2_O_3_)/Al designed for neutron absorbing materials by constructing lamellar structure. Compos. Part B Eng..

[8] Paidar M., Bokov D., Mehrez S., Ojo O.O., Ramalingam V.V., Memon S. (2021). Improvement of mechanical and wear behavior by the development of a new tool for the friction stir processing of Mg/B_4_C composite. Surf. Coat. Technol..

[9] Demirskyi D., Vasylkiv O. (2017). Analysis of the high-temperature flexural strength behavior of B_4_C–TaB_2_ eutectic composites produced by in situ spark plasma sintering. Mater. Sci. Eng. A.

[10] Zhang W. (2021). A review of tribological properties for boron carbide ceramics. Prog. Mater. Sci..

[11] Zhao X., Zou J., Ji W., Wang A., He Q., Xiong Z., Wang W., Fu Z. (2022). Processing and mechanical properties of B_4_C-SiCw ceramics densified by spark plasma sintering. J. Eur. Ceram. Soc..

[12] Jin X., Tang C., Li Q., Wang D., Ding X., Ran S. (2022). Densification and strengthening mechanism in spark plasma sintering of B_4_C–VB_2_ ceramics via carbide boronizing. Ceram. Int..

[13] Ren D., Deng Q., Wang J., Yang J., Li Y., Shao J., Li M., Zhou J., Ran S., Du S. (2018). Synthesis and properties of conductive B_4_C ceramic composites with TiB_2_ grain network. J. Am. Ceram. Soc..

[14] Tu R., Li N., Li Q., Zhang S., Zhang L., Goto T. (2016). Effect of microstructure on mechanical, electrical and thermal properties of B_4_C-HfB_2_ composites prepared by arc melting. J. Eur. Ceram. Soc..

[15] Yu H., Namini A.S., Shakeri M.S., Delbari S.A., Van Le Q., Cha J.H., Kim S.Y., Jang H.W., Lee S.-H., Swiatkowska-Warkocka Z. (2022). HRTEM study and mechanical properties of ZrB_2_–SiC composite: An insight into in-situ carbon formation over the SPS process. Int. J. Refract. Met. Hard Mater..

[16] Cheng X., He R., Qu Z., Ai S., Fang D. (2015). High temperature flexural strength of B_4_C–ZrB_2_ ceramic at 1000–1600 °C in air. Ceram. Int..

[17] Yang Q., Hwang C., Khan A.U., Domnich V., Gronske E.D., Haber R.A. (2019). Anisotropy and residual stress in B_4_C-ZrB_2_ eutectic. Mater. Charact..

[18] He R., Jing L., Qu Z., Zhou Z., Ai S., Kai W. (2015). Effects of ZrB_2_ contents on the mechanical properties and thermal shock resistance of B_4_C–ZrB_2_ ceramics. Mater. Des..

[19] Lin X., Ai S., Feng Y., Gao D., Guo X., Liu Y., Xie B., Gong H., Zhang Y. (2017). Fabrication and properties of in-situ pressureless-sintered ZrB_2_/B_4_C composites. Ceram. Int..

[20] Guo W., Wu L., You Y., Lin H., Zhang G. (2016). Three-step reactive hot pressing of B_4_C–ZrB_2_ ceramics. J. Eur. Ceram. Soc..

[21] Rehman S.S., Ji W., Fu Z., Wang W., Wang H., Asif M., Zhang J. (2015). In situ synthesis and sintering of B_4_C/ZrB_2_ composites from B_4_C and ZrH_2_ mixtures by spark plasma sintering. J. Eur. Ceram. Soc..

[22] Wachtman J.B., Cannon W.R., Matthewson M.J. (2009). Mechanical Properties of Ceramics.

[23] Li P., Ma M., Wu Y., Zhang X., Chang Y., Zhuge Z., Sun L., Hu W., Yu D., Xu B. (2021). Preparation of dense B_4_C ceramics by spark plasma sintering of high-purity nanoparticles. J. Eur. Ceram. Soc..

[24] Song Q., Zhang Z.-H., Hu Z.-Y., Yin S.-P., Wang H., Ma Z.-W. (2019). Microstructure and mechanical properties of super-hard B_4_C ceramic fabricated by spark plasma sintering with (Ti_3_SiC_2_+ Si) as sintering aid. Ceram. Int..

[25] Liu G., Chen S., Zhao Y., Fu Y., Wang Y. (2020). The effect of transition metal carbides MeC (Me = Ti, Zr, Nb, Ta, and W) on mechanical properties of B_4_C ceramics fabricated via pressureless sintering. Ceram. Int..

[26] Wang A., Hu L., Guo W., Zhao X., Shi Y., He Q., Wang W., Wang H., Fu Z. (2022). Synergistic effects of TiB_2_ and graphene nanoplatelets on the mechanical and electrical properties of B_4_C ceramic. J. Eur. Ceram. Soc..

[27] Liu G., Chen S., Zhao Y., Fu Y., Wang Y. (2021). Effect of Ti and its compounds on the mechanical properties and microstructure of B_4_C ceramics fabricated via pressureless sintering. Ceram. Int..

[28] Hwang C., DiPietro S., Xie K.Y., Yang Q., Celik A.M., Khan A.U., Domnich V., Walck S., Hemker K.J., Haber R.A. (2019). Small amount TiB_2_ addition into B_4_C through sputter deposition and hot pressing. J. Am. Ceram. Soc..

[29] Guo W., Wang A., He Q., Tian T., Liu C., Hu L., Shi Y., Liu L., Wang W., Fu Z. (2021). Microstructure and mechanical properties of B_4_C-TiB_2_ ceramic composites prepared via a two-step method. J. Eur. Ceram. Soc..

[30] Khajehzadeh M., Ehsani N., Baharvandi H.R., Abdollahi A., Bahaaddini M., Tamadon A. (2020). Thermodynamical evaluation, microstructural characterization and mechanical properties of B_4_C-TiB_2_ nanocomposite produced by in-situ reaction of nano-TiO_2_. Ceram. Int..

[31] Liu Z., Wang D., Li J., Huang Q., Ran S. (2017). Densification of high-strength B_4_C-TiB_2_ composites fabricated by pulsed electric current sintering of TiC-B mixture. Scr. Mater..

[32] Huang S., Vanmeensel K., Vleugels J. (2014). Powder synthesis and densification of ultrafine B_4_C-ZrB_2_ composite by pulsed electrical current sintering. J. Eur. Ceram. Soc..

[33] Zhu Y., Wang F., Wang Y., Cheng H., Luo D., Zhao Y. (2019). Mechanical properties and microstructure evolution of pressureless-sintered B_4_C–SiC ceramic composite with CeO_2_ additive. Ceram. Int..

[34] Roy A.M. (2020). Influence of interfacial stress on microstructural evolution in NiAl alloys. JETP Lett..

[35] Javanbakht M. (2021). High pressure phase evolution under hydrostatic pressure in a single imperfect crystal due to nanovoids. Materialia.

